# Prevalence of Staphylococcus Species Colonization in Healthy and Sick Cats

**DOI:** 10.1155/2019/4360525

**Published:** 2019-01-20

**Authors:** K. Bierowiec, A. Korzeniowska-Kowal, A. Wzorek, K. Rypuła, A. Gamian

**Affiliations:** ^1^Division of Infectious Diseases and Veterinary Administration, Department of Epizootiology and Clinic of Birds and Exotic Animals, Faculty of Veterinary Medicine, Wroclaw University of Environmental and Life Sciences, 50-366 Wroclaw, Poland; ^2^Department of Immunology of Infectious Diseases, Hirszfeld Institute of Immunology and Experimental Therapy, 53-114 Wroclaw, Poland

## Abstract

*Staphylococcus* is one of the most frequently isolated genera of opportunistic bacteria in animals and human beings. Staphylococci in mammals mostly inhabit the skin and mucous membranes. The objectives of the study were to investigate the distribution of staphylococcal species in healthy and sick cats in order to find diagnostic markers. The risk factors associated with colonization were also explored. Isolates from healthy (n=520) and sick cats (n=67) were identified at the species level using matrix-assisted laser desorption ionization time of flight mass spectrometry (MALDI-TOF MS). Swabs from conjunctival sacs, nares, skin, anus, and wounds were investigated using this technique. The diversity of the* Staphylococcus* species was high: 26 and 17 species in healthy and sick cats, respectively, and predominantly coagulase-negative staphylococci (CoNS) were isolated. The most frequently observed were* S. felis *and* S. epidermidis* in healthy cats, whereas* S. felis* and* S. haemolyticus* were most often found in sick animals.* S. aureus* strains were only isolated from healthy cats, whereas the only coagulase-positive* Staphylococcus* (CoPS) which occurred in the sick cats group was* S. pseudintermedius*. The sick, more frequently than the healthy animals, were colonized with* S. pseudintermedius* and* S. haemolyticus *and the relationship was statistically significant. Mostly, regardless of the state of their health, similar* Staphylococcus* species were isolated from cats; therefore, particular attention should be paid during the interpretation of diagnostic results.

## 1. Introduction

Animals are the natural habitats of complex populations of microorganisms. Between the host and microorganisms, homeostasis is produced which is one of the conditions for a proper and healthy functioning of the body [1]. Initially, during fetal life, the skin and mucous membranes remain sterile, but already during labor they are inhabited by many species of bacteria, which aids in protecting the body [2]. Continuous interactions between the organism and commensal microorganisms influence the development and regulation of the host's immune system [1]. Usually, the normal bacterial flora has a positive impact on the host, but in some circumstances, it can lead to the severe effects on the health state.

Due to the different ways in which the host and the bacterial microflora interact, we have been able to distinguish between the symbiotic, commensal, and opportunistic bacteria [3]. The opportunistic microflora is especially important because under favorable conditions, usually with a decline in the body's natural immunity or when it enters another organ of body, other than its regular habitat, it can cause an infection to develop. The species composition of the natural bacterial flora depends on the animal's species, way of feeding and living environments.

*Staphylococcus* is one of the most frequently isolated genera of opportunistic bacteria in animals and human beings. In mammals, staphylococci physiologically inhabit primarily the skin, mucous membrane of the nasal cavity, throat, and anus [4, 5]. In view of some diagnostic difficulties which are connected with opportunistic bacterial infections, profound knowledge about them is necessary. Baseline data on the species of bacteria found in various body sites can be of use in interpreting the importance of these bacteria in clinical samples. In clinical laboratory practice, it is often uncommon to identify* Staphylococcus* up to the species level for different reasons, such as high species variety, unclear taxonomic position, and unreliable identification results using biochemical tests. Therefore, there is a lack of data about the prevalence of certain staphylococci species in animals. This knowledge could be necessary during the diagnostic process because different Staphylococcus species might have varied pathogenicity mechanisms, pathogenesis, or peculiar transmission aspects [6].

The present study reports the prevalence of* Staphylococcus* colonization in cats including their state of health. The objective of the study was to systematically test the sensitivity of different anatomical locations and to identify risk factors for* Staphylococcus* colonization in cats by investigating certain characteristics of the animals and environment where they live.

## 2. Materials and Methods

### 2.1. Study Population and Sampling Procedures

Two groups of animals were examined: healthy cats and cats with clinical signs of conjunctivitis, upper respiratory tract disease, or skin/wound infection. The group of healthy cats was made up of two subgroups: animals that lived in households in close contact with their owners and free-living cats sampled during a trap, neuter, and release (TNR) program for the humane control of the feral cat population. The animals were included in the examination group after receiving permission from the owners to take samples of the cats. Additionally, each owner was asked to fill out a survey about the cat being examined and about the household. To assess the colonization with staphylococci in the animals under investigation, four swabs were taken from each one as follows: from the conjunctival sacs, nares, anus, and skin (groin). Additionally, in the group of sick cats an extra swab was collected from the wound or skin with pathological changes if any had occurred. The material was collected by a veterinary physician and placed into 2 ml of liquid brain-heart infusion broth (BHI) (Oxoid, United Kingdom). After the incubation of the material at 37°C for 24 hours, the samples were stored at –80°C in bacterial stock with 15% glycerol.

The research project was submitted to the 2^nd^ Local Ethics Committee for Animal Experiments in Wrocław. Due to the noninvasive samples collection procedure, the Ethics Committee qualified the study as research which therefore did not require any further approval from the Ethics Committee. Each cat owner consented to take part in this study and filled out the proper documentation.

### 2.2. Isolation and Identification of Staphylococcus Spp. from Samples

One microliter of bacterial glycerol stock was subcultured in Mannitol Salt Agar and blood agar plate (Oxoid, United Kingdom). The plates were then incubated for 24 hours. The incubation was extended to 48 hours if the result of the culture was negative or uncertain. The preliminarily identification of staphylococci was according the colony morphology, Gram staining, and detection of enzyme production (coagulase tube test; IBSS Biomed, Poland). Morphologically distinguishable staphylococcal colonies were cultured again in Mannitol Salt Agar and blood agar plate up to obtain pure cultures. A single colony from selected, pure strains was further identified by matrix-assisted laser desorption ionization time of flight mass spectrometry (MALDI-TOF MS) as previously described [7]. Raw spectra were processed using MALDI Biotyper v.3.1 software (Bruker Daltonik GmbH, Germany). Results were classified using score values proposed by the manufacturer.

### 2.3. Statistical Analysis

To calculate the prevalence and confidence intervals of staphylococcal species in each group, the two-step bootstrap method was used. In the first step, households (each with equal probability) were drawn from the pool of all households in the group. In the second step, one cat was drawn from each household. This process was repeated 10,000 times. The use of this method enabled the elimination of bias, which could have been the result of cats infecting each other in the same household.

The characteristics of the cats and questionnaire answers were compared to the staphylococcal species colonization scores. The data were analyzed using the chi-squared and Wilcoxon tests. P < 0.05 was considered to indicate a statistically significant association. Statistical analysis was carried out using the R Statistical Package (v. 2.11.1).

## 3. Results

A total of 587 cats were examined from 2013 to 2017 at the Department of Epizootiology and Clinic of Bird and Exotic Animals, Faculty Veterinary Medicine, Wrocław University of Environmental and Life Sciences, Poland. Cats were assigned to two groups based on data obtained from clinical examination and a diagnostic interview with the owner: healthy cats and sick cats (with at least one of the following clinical signs: conjunctivitis, upper respiratory tract disease, and skin or wound infection). Additionally, according to the surveys of 267 households, three subgroups were distinguished: single feline (only one purebreed or mixed breed cat in the household); multiple feline (more than one purebreed or mixed breed cat in the group, but not in the registered cattery); and cat breed (purebreed cats in a registered cattery kept in the same condition as pet cats (in households). In some cases, more than one cat was swabbed from the household. In addition, the healthy group of feral cats living in the urban area were swabbed. Detailed data from the animals under investigation are presented in Table 1.

**Figure 1:**
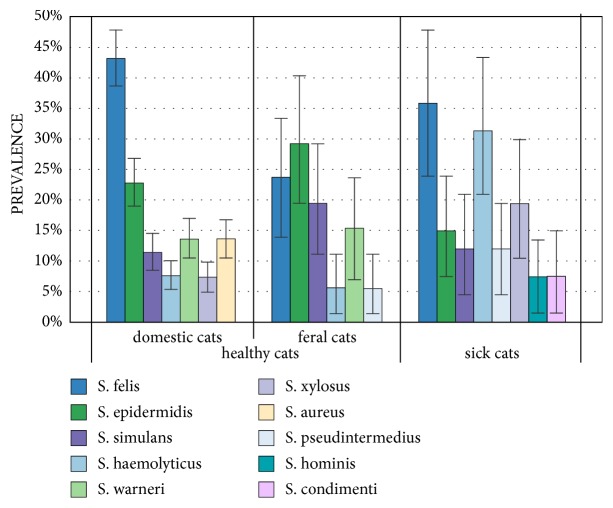
Prevalence of the most frequently isolated staphylococci from the cats under investigation.

At least one of the* Staphylococcus* species was isolated from 82.81%, 76.4% and 91% of healthy domestic cats, feral cats, and sick animals, respectively. Twenty-six different* Staphylococcus* species were isolated from healthy cats and 17 from sick animals. In the group of healthy cats, a higher diversity of the bacterial species was observed in animals kept in the households (24 species) than in feral cats (18 species).* S. epidermidis, S. felis, S. simulans, *and* S. warneri* were isolated from all the anatomical locations investigated in healthy cats, whereas* S. felis, S. simulans,* and* S. pseudintermedius *were isolated in sick cats. Detailed data about the average prevalence of the* Staphylococcus* species in the cats under investigation determined using the bootstrap method is presented in Table 2. Ten Staphylococcus species (shown in Figure 1) dominated in the cats under investigation and were isolated from more than 5% of animals. The most frequently observed were* S. felis *and* S. epidermidis* in healthy cats and* S. felis* and* S. haemolyticus *in the sick animals. The comparison of the most frequently isolated* Staphylococcus* species in both groups of cats is presented in Figure 1.

In the sick cats group, there were animals with the following clinical signs: conjunctivitis (n=7), sneezing (n=2), conjunctivitis and sneezing (n=50), and wound infection (n=8). Half of the cats under investigation (50.75%) were colonized with the staphylococci in the location with pathological changes and 67.65% of the cats were colonized with the same* Staphylococcus* species in another anatomical location as well. Detailed data about the distribution of the* Staphylococcus* species in sick cats are presented in Table 3.

Statistical analysis confirmed that the animals in the group of sick cats were more frequently colonized with* S. pseudintermedius *and* S. haemolyticus*. Such features as breed, age, and sex did not have any influence on the occurrence of* Staphylococcus* species. Among the features of the households, where the cats were kept, the following turned out to be the most important: the number of household residents, occupation of the family members, and the number of animals kept in the same households. There were all contributing factors. No correlation was observed between the colonization of specific species and anatomical location. Detailed data of statistical analysis are shown in Table 4.

## 4. Discussion

This study provides detailed data about* Staphylococcus* carriage in healthy and sick cats. The identification of staphylococci was carried out by MALDI-TOF MS, which provides reliable and rapid identification of the taxa in the* Staphylococcus* genus, including the* Staphylococcus* Intermedius Group (SIG). Cats have been implicated as carriers of both coagulase-positive (CoPS) and coagulase-negative* Staphylococcus* species (CoNS) [8]; nevertheless, according to the results of our study, the occurrence of respective staphylococcal species was different in sick and healthy animals.

A similar study showed that* Staphylococcus* is more frequently isolated from sick cats than from healthy ones [9]. Our study confirmed the result, although the differences in the percentage of colonized animals in both groups are minor and they were not statistically significant. The spectrum of staphylococcal species in healthy domestic cats was wider than in feral or sick cats. Gandolfi-Decristophoris et al. [8] also reported such variety of bacterial species in animals that lived in the community. Places on the body which showed pathological changes were dominated by CoNS, in contrast to findings of other studies, where usually* S. pseudintermedius* or* S. aureus* were isolated [10, 11]. The lack of* S. aureus *isolates among materials collected from sick animals was surprising, whereas the species was the most frequently isolated CoPS in the healthy animals group. The result could confirm the hypothesis that* S. aureus *usually constitute the natural bacterial flora in cats, especially in animals kept in close contact with their owners [12]. Another reason for such a situation might be the difficulty of the distinction between S. aureus and* S. pseudintermedius* using standard laboratory methods and wrong classification of some* S. pseudintermedius* strains as* S. aureus* strains. Correlating the results of our study with other reports, CoPS species such as* S. pseudintermedius *and* S. schleiferi *spp*. coagulans* are more typical for healthy dogs than cats [8, 11, 13, 14] and in cats isolation of the staphylococci may testify to a bacterial infection. Moreover,* S. pseudintermedius* was statistically and significantly more frequently isolated from the sick cat group in our study. The occurrence of the bacteria in healthy cats was similar to results of other studies [13, 15], but the comparison of results of colonization in sick cats is not possible because there is still a lack of such studies. The frequency of isolation of* S. pseudintermedius* from sick cats ranges from 2,1% [16] to 7,9% [11] whereas the incidence of methicillin-resistant S*. pseudintermedius* (MRSP) strains was from 2% [16] to 10%–13,5% [6, 17].

Regardless of the health state in all groups,* S. epidermidis, S. felis,* and* S. simulans* were the most frequently isolated. Despite the isolation of the bacteria also from pathologically changing locations, in most cases the pathogens constitute rather the natural microbiota than the real reason of the infection, because the species were equally frequently isolated in the same locations in both healthy and sick cats. Nevertheless, there are reports about severe infection in animals and humans caused by such CoNS species [18–20] and they constitute a potential risk for the health of immunocompromised individuals.

*S. haemolyticus* is one of the most frequently reported CoNS species in humans and has been associated with septicemia, endocarditis, peritonitis, and wound, joint, and bone infections [21]. The bacteria are highly prevalent in the hospital environments and its resistance to multiple antibiotics has often been observed [22]. In our study, a significant number of animals were colonized with* S. haemolyticus*, and it was the reason for the majority of cases of the upper respiratory tract infections. It was also one of the* Staphylococcus* species which was, statistically, more frequently encountered in sick animals group.* S. haemolyticus* is often isolated from pets as well as from farm animals; however, mainly the reports focus only on methicillin-resistant strains [6, 23].

Among staphylococci isolated in this study, only* S. carnosus* was not previously reported in pets [8]. This microorganism is used as a starter culture in food fermentation and widely known as a harmless species [24] and may be dismissed as an insignificant contaminant when isolated via bacterial culture. The* Staphylococcus* which deserves more attention in pet animals is* S. lugdunensis* which has been assumed to be nonpathogenic to companion animals and have been often excluded as the potential cause of infection [25]. In pets similarly to people,* S. lugdunensis *is known to cause severe, deep soft tissue infections. However this species does not possess secreted coagulase, but some isolates have an ability to produce a clumping factor which may be the reason of misclassification as* S. aureus* in a standard laboratory diagnostic [25, 26]. This bacterium may not show a high virulence level similar to* S. aureus,* but its virulence is higher than that of all other CoNSs [27].

This study provides a comprehensive investigation of risk factors for the colonization of cats with different* Staphylococcus* species. Although for a limited number of species for which such an analysis was possible, we identified some risk factors: the higher number of the household's members for* S. aureus* and* S. equorum*; one or more owners working in healthcare or in veterinary healthcare for* S. aureus*; and dogs or other animals being kept with the cat under investigation for* S. felis*,* S. equorum,* and* S. nepalensis*. Usually, reports focused on the risk factors in dogs population. As for now, only the risk factors for* S. aureus* and* S. pseudintermedius* colonization were investigated in cats [28–30]. The main risk connected with* S. aureus* colonization raised from the number of antimicrobial courses, hospitalization, and surgery in dogs and cats [8, 28] whereas the risk of* S. pseudintermedius* colonization was associated with hospitalization, frequent visiting of veterinarians, and admission of glucocorticosteroids [30]. Our study has not shown such a relationship, but we did not have access to the treatment history of the cats under investigation and the data were obtained directly from the owners; therefore, a recall bias might be present. Nevertheless, we report the significantly higher prevalence of* S. haemolyticus *and* S. pseudintermedius* in sick cats group what partly might be connected with the previous visiting of the veterinary clinic and treatment. The influence of the owners' occupation is discussed, and some reports confirmed such a risk [31, 32]; our study has also shown this correlation. Similarly to previous reports, we did not find the influence of age, breed, or sex on the colonization with* Staphylococcus* [28]. Because of a gaining significance of other* Staphylococcus*,* S. pseudintermedius,* and* S. haemolyticus* among others, future studies should also focus on their risk factors.

It should be noted that our study has some limitations. All data were collected at a single point in time; therefore, the length of colonization could not be investigated. Furthermore, in some cases, the risk factor assessment was not possible, in view of a low number of colonized cats under investigation with some* Staphylococcus* species. Some other limitations could be associated with the pet management factor in the one year preceding the study which was reported by the owners.

## 5. Conclusion

This study confirmed that skin, nares, conjunctival sacs, and anus in cats are mainly colonized due to staphylococci. The knowledge about the natural bacterial flora of specific individuals is necessary for the proper interpretation of diagnostic results and estimation of the risk associated with the colonization by a specific microorganism.* S. aureus* strains were only isolated from healthy cats, whereas the only coagulase-positive staphylococci which occurred in the sick cats' group was* S. pseudintermedius*. Furthermore, the risk of colonization with* S. pseudintermedius* and* S. haemolyticus* is significantly higher in sick animals than in healthy ones. This is useful information to guide clinical decision and future studies.* Staphylococcus* species are especially important to human and animal health; therefore, future studies should address the duration of colonization in pets and the possible transmission of* Staphylococcus* across species.

## Figures and Tables

**Table 1:** Demographic characteristics of the investigated cats.

	Category	Number of investigated households	Number of cats	Sex		Breed		Age								
				Female (%)	Male (%)	Crossbreed (%)	Breed (%)	≤6 (month)			7–36 (month)			≥37 (month)		
								%	x−	*σ*	%	x−	*σ*	%	x−	*σ*
Healthy cats	Multiple feline	101	150	48.00	52.00	72.00	28.00	8.00	4.67	1.61	47.33	18.65	9.07	44.67	82.03	39.35
	Single feline	116	116	54.31	45.69	60.34	39.66	10.34	5.08	1.31	62.07	17.54	9.30	27,59	95.03	43.44
	Purebreed cats	18	182	60.99	39.01	-	100	24.18	2.91	1.12	64.84	22.35	9.70	10.99	70.91	28.05
	Feral cats	-	72	69.4	30.6	100	-	5.56	5.75	0.5	91.67	16.12	843	27.78	48.00	0

Sick cats	Multiple feline	13	30	46.67	53.33	86.67	13.33	46.67	4.43	1.50	33.33	29.80	7.33	20.00	73.33	35.14
	Single feline	17	17	52.94	47.06	58.82	41.18	35.29	2.33	1.86	52.94	16.44	7.72	11.76	60.00	0
	Purebreed cats	2	20	65.00	35.00	-	100	70.00	3.55	1.21	25.00	24.8	9.01	5.00	48	0

x: arithmetic mean.

*Σ: *standard deviation.

**Table 2:** Prevalence of Staphylococcus species in healthy and sick cats.

Species	n	Healthy cats								Sick cats				
		Skin %*∗* (CI)		Nares %*∗* (CI)		Conjunctival sacs %*∗* (CI)		Anus %*∗* (CI)		Skin %*∗* (CI)	Nares %*∗* (CI)	Conjunctival sacs %*∗* (CI)	Anus %*∗* (CI)	Wound %*∗* (CI)
		DC	FC	DC	FC	DC	FC	DC	FC					
*S. arlettae*	3	0.42 (0-1.28)				0.25 (0-0.85)								

*S. aureus*	86	4.24 (2.46-6.25)		7.37 (4.27-10.68)		5.79 (2.99-9.40)	2.80 (0-6.94)	2.89 (0.85-5.23)						

*S. capitis*	21	0.89 (0-2.14)	1.38 (0-4.17)	2.05 (0.43-4.27)		1.64 (0.43-3.42)		<0.01	1.38 (0-4.17)				0.22 (0-3.03)	

*S. caprae*	5			0.24 (0-0.85)		0.21 (0-0.85)		0.04 (0-0.43)				3.18 (0-9.09)		

*S. carnosus*	1							0.42 (0-1.28)						

*S. cohnii*	12	0.44 (0-1.71)			1.37 (0-4.17)	0.85 (0-2.14)	1.40 (0-4.17)	0.42 (0-1.28)						

*S. condimenti*	31	0.58 (0-1.71)		1.32 (0-3.00)	1.38 (0-4.17)	1.87 (0.43-3.85)	2.79 (0-6.94)	0.48 (0-1.71)		3.09 (0-9.09)	4.17 (0-12.12)	1.25 (0-6.06)	0.21 (0-3.03)	

*S. epidermidis*	175	8.52 (5.13-12.39)	12.52 (5.56-20.83)	4.85 (2.56-7.70)	8.30 (2.78-15.28)	10.20 (6.41-14.53)	11.1 (4.17-19.44)	1.96 (0.43-3.85)	2.76 (0-6.94)	7.46 (1.49-14.93)	0.19 (0-3.03)	8.69 (0-18.18)		2.87 (0-9.09)

*S. equorum*	22	0.53 (0-1.71)	1.39 (0-4.17)	2.14 (0.43-4.27)		0.93 (0-2.14)		0.87 (0-2.14)			0.023 (0-3.03)	0.22 (0-3.03)		

*S. felis*	368	5.67 (2.99-8.97)	1.38 (0-4.17)	22.13 (17.09-27.35)	13.88 (6.94-22.22)	13.50 (8.97-17.95)	12.46 (5.56-20.83)	15.58 (11.11-20.51)	2.80 (0-6.94)	17.43 (6.06-30.30)	35.82 (21.21-54.55)	23.95 (12.12-39.39)	19.71 (6.06-33.33)	2.87 (0-9.09)

*S. haemolyticus*	81	1.98 (0.43-3.85)	1.38 (0-4.17)	2.08 (0.43-3.84)		3.35 (1.28-5.56)	1.38 (0-4.17)	0.90 (0-2.14)	2.78 (0-6.94)	7.57 (0-18.18)	7.49 (0-18.18)	4.84 (0-12.12)	0.62 (0-3.03)	

*S. hominis*	23	0.22 (0-0.85)	2.79 (0-6.94)	1.63 (0.43-3.42)		1.08 (0-2.56)	1.35 (0-0-4.17)	0.67 (0-1.71)		3.07 (0-9.09)	9.94 (0-12.12)			

*S. lentus*	6	0.02 (0-0.43)	1.41 (0-4.17)	0.45 (0-1.70)		0.42 (0-1.28)	1.39 (0-4.17)							

*S. lugdunensis*	5			0.09 (0-0.43)	1.40 (0-4.17)			0.86 (0-2.14)				0.14 (0-3.03)		

*S. nepalensis*	8	0.57 (0-1.71)		0.052 (0-1.70)		0.08 (0-0.43)		0.15 (0-0.85)						

*S. pasteuri*	7	0.87 (0-2.14)	2.77 (0-6.94)	0.19(0-0.85)		0.42 (0-1.23)					0.17 (0-3.03)			

*S. pettenkoferi*	4					0.57 (0-1.70)								

*S. piscifermentans*	1					0.42 (0-1.28)								

*S. pseudintermedius*	29	0.45 (0-1.23)		0.51 (0-1.7)	4.14 (0-9.72)	0.90 (0-2.14)	2.77 (0-6.94)	0.89 (0-2.14)		9.16 (0-21.21)	4.45 (0-10.45)	7.24 (0-18.18)	3.24 (0-9.09)	2.97 (0-9.09)

*S. saprophyticus*	19	1.05 (0-2.56)		1.08 (0-2.56)		1.66 (0.43-3.42)		0.68 (0-1.71)				3.09 (0-9.09)		

*S. schleiferi spp. coagulans*	1							0.43 (0-1.23)						

*S. sciuri*	20	0.63 (0-1.71)		2.05 (0.43-4.27)	1.41 (0-4.17)	<0.01		0.02 (0-0.43)		2.88 (0-9.09)	6.06 (0-15.15)			

*S. simulans*	94	5.32 (2.56-8.12)	2.77 (0-6.94)	6.15 (3.42-9.40)	12.53 (5.56-20.83)	2.90 (1.34-4.46)	5.52 (1.39-11.11)	2.64 (0.85-4.70)	2.79 (0-6.94)	6.22 (0-15.15)	9.17 (0-21.21)	6.11 (0-15.15)	0.96 (0-6.06)	3.07 (0-9.09)

*S. vitulinus*	18	1.27 (0-2.99)		1.75 (0.43-3.42)	1.38 (0-4.17)	1.29 (0-2.99)		1.73 (0.43-3.42)			0.20 (0-3.03)			

*S. warneri*	90	9.18 (5.56-13.25)	4.20 (0-9.72)	3.95 (1.71-6.41)	2.77 (0-6.94)	8.29 (4.70-11.97)	9.72 (2.78-16.67)	1.23 (0-2.99)	1.41 (0-4.17)	0.97 (0-6.61)	3.02 (0-9.09)	1.41 (0-6.06)		

*S. xylosus*	56	1.97 (0.43-3.85)		3.23 (1.28-5.56)	1.41 (0-4.17)	1.84 (0.43-3.85)		0.87 (0-2.14)		6.25 (0-9.09)	10.88 (3.03-21.21)	10.49 (0-21.21)	3.30 (0-9.09)	

n: number of isolated strains.

DC: domestic cats.

FC: feral cats.

CI: confidential interval 95%.

**Table 3:** Comparison of *Staphylococcus* species which were isolated from sick animals.

Clinical signs	Anatomical location	Staphylococcus species (*∗*)
Conjunctivitis and sneezing	Conjunctival sacs	*S. condimenti* (2%) *S. epidermidis* (2%) *S. equorum* (2%) *S. felis* (12%) *S. haemolyticus *(26%) *S. lugdunensis* (2%) *S. simulans *(2%) *S. xylosus* (2%)
	Nares	*S. condimenti* (4%) *S. epidermidis* (2%) *S. equorum* (2%) *S. felis* (20%) *S. haemolyticus* (18%) *S. hominis* (6%) *S. pasteuri* (2%) *S. pseudintermedius* (4%) *S. sciuri *(4%) *S. simulans* (4%) *S. vitulinus* (2%) *S. xylosus *(4%)

Sneezing	Nares	*S. condimenti *(50%) *S. felis* (50%)

Conjunctivitis	Conjunctival sacs	*S. caprae* (14%) *S. felis* (14%) *S. pseudintermedius* (28,5%) *S. warneri* (14%) *S. xylosus* (43%)

Wound infection	Wound	*S. epidermidis* (12,5%) *S. felis *(12,5%) *S. pseudintermedius *(12,5%) *S. simulans* (12,5%)

*∗* The percentage of cats with designated clinical signs colonized with the staphylococcal species.

**Table 4:** Statistical analysis results of risk factors associated with the colonization of Staphylococcus in cats under investigation.

Variable	Test	Species	P value	OR	95%CI
State of health	Chi-squared*∗*	*S. haemolyticus* *S. pseudintermediu*s	0 0.02856	5.54 4.93	2.93-10.32 1.84-12.56

Breed	Chi-squared*∗*	all tested species	> 0.05		

Age	Wilcoxon	all tested species	> 0.05		

Sex	Chi-squared*∗*	all tested species	> 0.05		

Number of households residents who had close contact with the cat under investigation	Wilcoxon	*S. aureus* *S. equorum*	0.0252 0		

Family member works in healthcare or in veterinary healthcare	Chi-squared*∗*	*S. aureus*	0.017	2.66	1.54-4.60

Hospitalization of an owner in the previous year	Chi-squared*∗*	all tested species	>0.05		

Diagnosis of Staphylococcus colonization in the previous year: in the household resident In the cat under investigation	Chi-squared*∗*	all tested species	>0.05		

Number of animals kept in the same household					

Dogs	Wilcoxon	*S. nepalensis*	0.028		

Cats	Wilcoxon	all tested species	>0.05		

Others	Wilcoxon	*S. equorum* *S. felis*	0 0.028		

Treatment of cat under investigation in the previous year	Chi-squared*∗*	all tested species	> 0.05		

Treatment of other pets in the previous year	Chi-squared*∗*	all tested species	> 0.05		

P value: probability value; Chi-squared*∗*: degrees of freedom is 1; OR: odd ratio; CI: confidential interval.

## Data Availability

The data used to support the findings of this study are available from the corresponding author upon request.
